# Advancing Neuroblastoma Surgery through the Clinical Integration of Virtual Reality and Indocyanine Green Fluorescence-Guided Imaging: A Case Report

**DOI:** 10.1055/a-2646-8880

**Published:** 2025-07-24

**Authors:** Irene Paraboschi, Ugo M. Pierucci, Elena Di Blasi, Paola Collini, Marta Podda, Giovanna Gattuso, Roberto Luksch, Francescco Rizzetto, Alice M. Munari, Cristina Gallotta, Tommaso Santaniello, Maurizio Vertemati, Paolo Milani, Gloria Pelizzo

**Affiliations:** 1 Department of Biomedical and Clinical Science, University of Milan, Milan, Lombardy, Italy; 2 Department of Pediatric Surgery, “V. Buzzi” Children's Hospital, Milano, Italy; 3 Soft Tissue Tumor Pathology Unit, Department of Advanced Diagnostics, Fondazione IRCSS Istituto Nazionale dei Tumori, Milano, Italy; 4 Pediatric Oncology Unit, Fondazione IRCCS Istituto Nazionale Dei Tumori, Milano, Italy; 5 Department of Radiology, ASST Grande Ospedale Metropolitano Niguarda, Milano, Italy; 6 Postgraduate School of Diagnostic and Interventional Radiology, University of Milano, Milano, Italy; 7 Department of Pediatric Radiology, “V. Buzzi” Children's Hospital, Milano, Italy; 8 CIMaINa - Interdisciplinary Centre for Nanostructured Materials and Interfaces, University of Milano, Milano, Italy; 9 Department of Physics “Aldo Pontremoli,” University of Milano, Milano, Italy

**Keywords:** neuroblastoma, virtual reality, indocyanine green

## Abstract

**Background:**

Neuroblastoma, the most common extracranial solid tumor in children, requires meticulous surgical interventions due to its complex anatomical location and proximity to vital structures. Emerging technologies, such as virtual reality (VR) and indocyanine green (ICG) fluorescence-guided imaging, offer promising solutions to enhance surgical precision and outcomes. Despite their potential, their use in pediatric oncology remains underexplored. This case report highlights the integration of VR and ICG fluorescence imaging in the surgical treatment of neuroblastoma, emphasizing their benefits, limitations, and the need for further advancements.

**Case Description:**

A 12-month-old female with a prenatal diagnosis of cloacal malformation, Müllerian anomalies, and a horseshoe kidney was under care at our center for the management of her complex urogenital anomalies. During preoperative imaging to plan her reconstructive surgery, an abdominal MRI revealed a solid retroperitoneal mass, later confirmed as a right adrenal neuroblastoma. After six cycles of chemotherapy, metaiodobenzylguanidine (mIBG) scans indicated persistent uptake, suggesting the possible presence of tumor viability. Consequently, a definitive surgical resection was scheduled. The procedure incorporated VR for navigation and ICG fluorescence for real-time vascular mapping, facilitating precise dissection and preservation of critical structures. The patient's postoperative recovery was uneventful, and she was discharged in stable condition. Follow-up evaluations (i.e., MRI, mIBG) showed no evidence of residual macroscopic disease.

**Conclusion:**

VR and ICG fluorescence imaging hold promise for enhancing surgical precision and safety in pediatric neuroblastoma. While current limitations include the lack of real-time image overlay and inadequate visualization of tumor margins, future advancements in navigation systems and targeted probes may overcome these barriers and significantly improve oncologic outcomes.

## Introduction

Neuroblastoma is a pediatric malignancy accounting for approximately 8 to 10% of all childhood cancers. 1 2 Originating from neural crest cells, these tumors are often located in the adrenal glands or along the sympathetic chain, making surgical resection particularly challenging. 1 2 The primary surgical goal is the complete removal of the tumor while preserving critical surrounding structures, such as major blood vessels and organs. Traditional surgical approaches rely heavily on preoperative imaging modalities like MRI and CT scans, and the surgeon's intraoperative judgment. 3 However, these methods can be limited by the surgeon's ability to translate static images into the dynamic surgical field.

Emerging technologies such as virtual reality (VR) and fluorescence-guided surgery (FGS) offer promising avenues to enhance surgical accuracy. VR enables real-time integration of preoperative imaging data into the operative field, improving spatial awareness and anatomical precision. 4 5 6 7 8 Indocyanine green (ICG) fluorescence, widely used for vascular mapping, offers additional intraoperative insights into tissue perfusion and vascular anatomy. 9 10 11 12 13 Although these technologies are increasingly used in adult surgeries, 14 15 16 17 18 their application in pediatric oncology remains limited. 6 7 8 9 12 13 VR and ICG-based FGS are increasingly used by surgeons. ICG-based FGS has been used to enhance anatomical visualization, guide resections, and improve intraoperative decision-making, especially in hepatobiliary and colorectal procedures. 14 15 16 17 18 These technologies provide real-time feedback that supports margin assessment and vascular mapping, helping to reduce complications. 14 15 16 17 18 Their successful application in adult cases reinforces their potential value in complex pediatric oncologic surgery. 6 7 8 9 This report presents a successful case of neuroblastoma resection using the combined modalities of VR and ICG fluorescence, highlighting their clinical impact and potential for widespread adoption.

## Case Presentation

**Video 1** Advancing Neuroblastoma Surgery through the Clinical Integration of Virtual Reality and Indocyanine Green Fluorescence-Guided Imaging: A Case Report


**Fig. 1:**
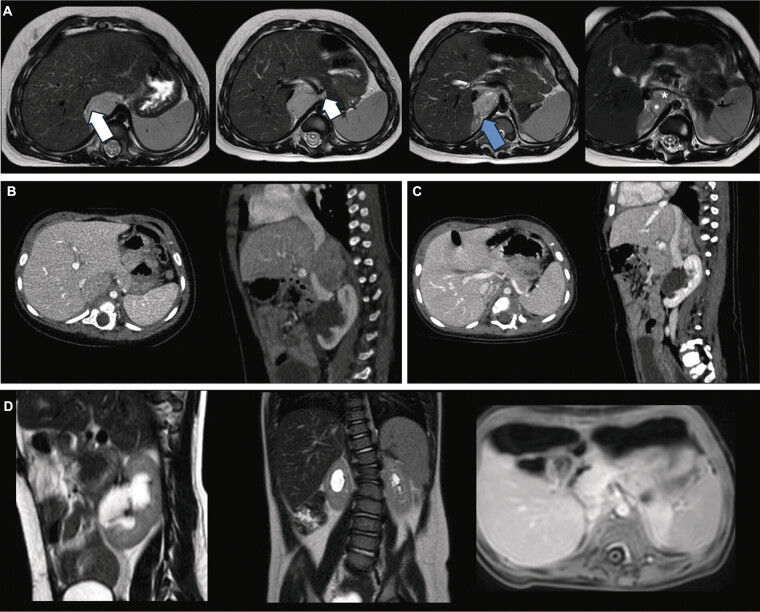
( **A** ) Axial T2-weighted MRI images at diagnosis showed a retroperitoneal mass closely related to the posterior hepatic margin and right diaphragmatic crus, adherent to the body of the right adrenal gland (blue arrow), inseparable from the compressed inferior vena cava (filiform intrahepatic lumen; long white arrow), extending to the midline and inter aortocaval space with encasement of the celiac trunk and branches (short white arrow), and contacting the left renal vein (*). ( **B** ) Abdominal CT scan. The imaging confirmed the presence of a solid, expansile lesion measuring approximately 3.5 × 2.5 × 4.5 cm (anteroposterior [AP] × laterolateral [LL] × craniocaudal [CC]) with an oval shape and heterogeneous density located in the right retroperitoneal, subdiaphragmatic region. The lesion was inseparable from the right adrenal gland and the ipsilateral diaphragmatic crus. It compressed the intrahepatic segment of the inferior vena cava, reducing its caliber to a minimum of 4 mm, though the vessel remained patent. The lesion extended into the interaortocaval space, approaching the midline, with no clear cleavage planes identified between the lesion and the hepatic artery, portal vein, right renal artery, pancreas head, upper pole of the right kidney, and left renal vein. Enlarged lymph nodes, with short axes measuring approximately 1 cm, were noted in the periaortic, interaortocaval, and iliac regions. ( **C** ) Preoperative abdominal CT scan. There was a substantial dimensional reduction of the previously identified solid retroperitoneal lesion, now measuring approximately 1 × 2.5 × 4 cm (AP × LL × CC). The lesion was in contact with the right adrenal gland, the right diaphragmatic pillar, the posterior hepatic profile in segments VI to VII, the left renal vein, and the inferior vena cava (without evidence of encasement). The mass was also near the superior polar/accessory right renal artery and a diaphragmatic artery originating from the aorta to the left of the celiac trunk. ( **D** ) Sagittal and coronal T2-weighted MRI images, along with axial T2-weighted and T1-weighted fat-saturated postcontrast images following the intervention, demonstrate that the previously expanded right retroperitoneal mass is no longer identifiable.

**Fig. 2:**
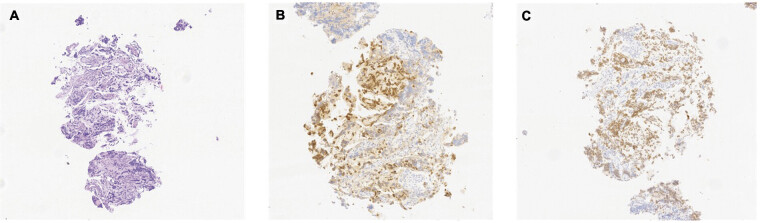
Histopathological findings of the laparoscopic biopsy. ( **A** ) Hematoxylin and eosin stain; ( **B** ) anti-Hu immunohistochemistry; and ( **C** ) anti-paired-like homeobox 2b (PHOX2B) immunohistochemistry.

A 12-month-old female with a prenatal diagnosis of cloaca malformation associated with Müllerian anomalies and a horseshoe kidney was followed at our center for the management of her complex urogenital anomalies. During preoperative imaging studies to plan the reconstructive gastro- and urogenital surgery, an abdominal MRI identified a solid retroperitoneal mass measuring 40 × 35 × 34 mm. The mass was located caudal to the diaphragm and closely adherent to the right adrenal gland. It predominantly exhibited homogeneous signal characteristics, with a small cystic component in its caudal portion, early enhancement, and marked diffusion restriction. The mass compressed the inferior vena cava (IVC), resulting in a filiform lumen in the intrahepatic tract. It extended into the midline and interaortocaval space, encasing the celiac trunk and its branches. Adjacent lymphadenomegaly was observed in multiple regions, including the hepatic hilum, retrocrural space, and para-aortic areas. These findings raised a high suspicion of a neuroblastic tumor ( Fig. 1A ). Subsequent CT imaging confirmed the presence of an oval-shaped, solid mass inseparable from the right adrenal gland, diaphragmatic crus, and surrounding critical structures, including the hepatic pedicle, renal vasculature, and IVC. The mass lacked definitive cleavage planes with these structures, reinforcing the suspicion of neuroblastoma ( Fig. 1B ). The patient underwent a laparoscopic biopsy, which confirmed the diagnosis of a poorly differentiated neuroblastoma originating from the right adrenal gland ( Fig. 2 ).

Due to the sample's limited cellularity, only the *N-MYC* gene status analysis could be performed, while a complete genomic characterization of the tumor was unfeasible. Bone marrow aspirates and an mIBG scan showed no metastasis evidence; the stage was classified as L2 according to the International Neuroblastoma Risk Group Staging System. 19

Primary surgery was deemed unfeasible due to the presence of image-defined risk factors related to the tumor's involvement with adjacent anatomical structures. Consequently, the patient underwent four cycles of chemotherapy (2 × carboplatin/etoposide, 2 × cyclophosphamide/doxorubicin/vincristine) according to the SIOPEN-LINES guidelines. 20 Posttreatment MRI after the four cycles still demonstrated inoperability, leading to the administration of an additional two cycles of chemotherapy (1 × carboplatin/etoposide, 1 × cyclophosphamide/doxorubicin/vincristine).

**Fig. 3:**
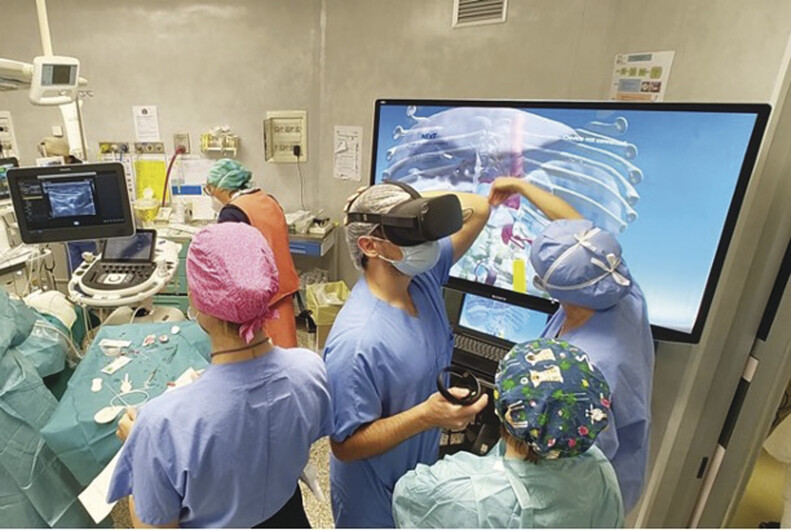
The image captures the dynamic operating room setting where surgeons, registrars, and scrub nurses collaborate closely around a cutting-edge VR model. This scene underscores the transformative integration of VR and advanced imaging technologies into modern surgical environments, emphasizing their potential to improve precision, optimize outcomes, and elevate the educational experience for health care teams. VR, virtual reality.

Despite systemic treatment, re-evaluation revealed the lesion's persistence on CT, with unchanged anatomical relationships ( Fig. 1C , 1D ), and high persistence of tumor uptake of guanidine on the mIBG scan. Given these findings and, most of all, the incomplete molecular characterization of the tumor, the patient underwent a definitive surgical resection. During the procedure, the surgical team employed a novel approach integrating VR and ICG fluorescence imaging ( Fig. 3 ). Preoperative CT data were processed using a semiautomatic segmentation approach, followed by manual corrections, to generate accurate 3D surface models of the malformations and associated anatomical structures. An expert radiologist subsequently reviewed these 3D models to verify the correspondence with the original CT images. The initial segmentation of key organs and vascular structures was performed using software-assisted tools, followed by manual corrections to ensure anatomical accuracy. This hybrid approach enables greater precision than fully automatic methods, particularly in pediatric patients with anatomical variability or congenital anomalies. Image segmentation and 3D model creation were performed using 3D Slicer, an open-source software platform for medical image computing. The entire process (from image import to finalized 3D model) required approximately 24 hours per case, depending on anatomical complexity. Upon approval, the 3D models were uploaded into a custom-developed VR environment and modified for use with the Oculus Quest 2 (META Inc., Menlo Park, CA) head-mounted display (HMD). 5 21 22 This setup provided the surgical team with an immersive visualization of the 3D model. 5 21 22 Using the wireless controller, the operator could navigate and interact with the 3D reconstruction in an immersive way. Functions included selecting different 3D structures, adjusting the transparency of various components, and rotating or zooming in and out of the 3D scene. During preoperative planning, surgeons wore the HMD, launched the application using the wireless controllers, selected a reconstruction, and explored it interactively. 5 21 22 The VR goggles (Oculus Quest 2) were used only before the surgery, during the preoperative planning phase. The immersive session involved all key surgical team members, including the primary operator, assistants, and scrub nurse, to ensure shared spatial awareness and procedural coordination. The Oculus Quest 2 is a non-transparent (blind) system, meaning it immerses the user in a virtual environment and does not allow visualization of the real surgical field. For this reason, it was not used intraoperatively. 5 21 22 Moreover, VR permitted navigation for a better intraoperative definition of the tumor. 5 21 22

**Fig. 4:**
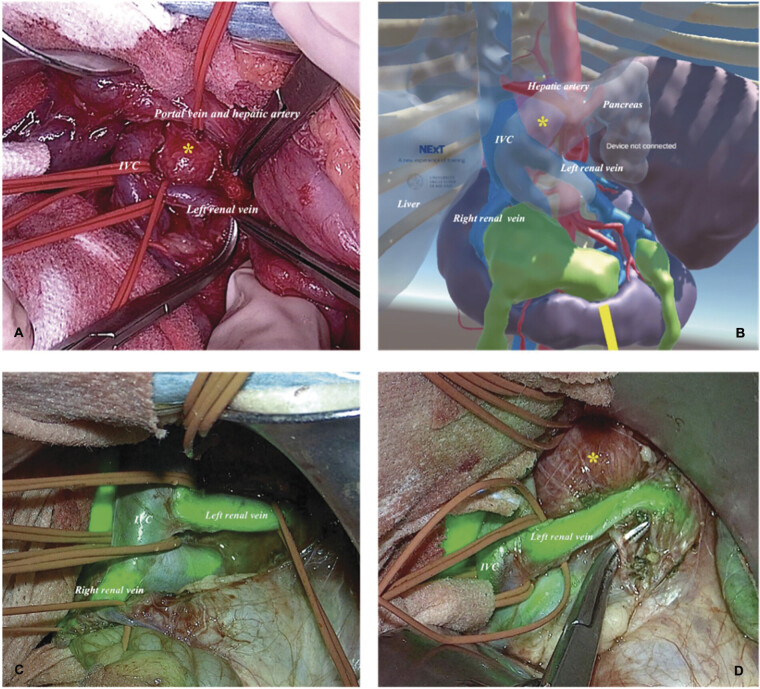
The image illustrates a combination of intraoperative findings ( **A** ), a VR 3D anatomical model ( **B** ), and ICG-based fluorescence-guided surgery ( **C** , **D** ), offering a comprehensive view of the surgical anatomy and vascular structures encountered during the procedure. The top-left panel ( **A** ) depicts the real-life surgical dissection, showcasing key vascular structures, including the portal vein, hepatic artery, inferior vena cava (IVC), left renal vein, and the neoplastic lesion (*). Adjacent to this, the top-right panel ( **B** ) presents a VR 3D anatomical model, visually representing the abdominal organs and vascular anatomy, such as the hepatic artery, IVC, left renal artery, and right renal artery, correlating with the intraoperative findings. The bottom-left panel ( **C** ) highlights the use of fluorescence imaging with ICG, significantly enhancing the visualization of vascular structures. The IVC, left renal artery, and right renal artery were distinctly illuminated, demonstrating the benefits of fluorescence guidance in surgery. Similarly, the bottom-right panel ( **D** ) continues to utilize fluorescence imaging, focusing on the IVC and left renal artery, highlighted in green, to provide precise anatomical visualization that facilitated the dissection process. However, as shown in the lower row ( **C** , **D** ), the neuroblastoma tumor did not fluoresce due to the lack of tumor-specific dye uptake. ICG, indocyanine green; VR, virtual reality.

ICG (0.15 mg/kg) was administered intravenously three times during the operation to enhance vascular visualization. Intraoperative fluorescence imaging was performed using the Rubina lens system (KARL STORZ SE & Co. KG, Tuttlingen, Germany), which enabled real-time visualization of vascular structures following intravenous administration of ICG. Isolating the neoplastic mass proved challenging due to its proximity to surrounding vascular structures, including the IVC, right and left renal veins, portal vein, and hepatic artery, as well as adjacent solid abdominal organs such as the pancreas and horseshoe kidney. In this complex scenario, the dye facilitated real-time vasculature imaging, ensuring the preservation of critical vessels such as the right renal vein and its vascular branches ( Fig. 4 ).

During the operation, VR and ICG fluorescence integration enabled meticulous and precise tumor dissection. The VR system provided continuous guidance, while the ICG fluorescence highlighted vascular structures, minimizing the risk of accidental injury or excessive bleeding.

Postoperative recovery was uneventful, and the patient was discharged on postoperative day 8.

**Fig. 5:**
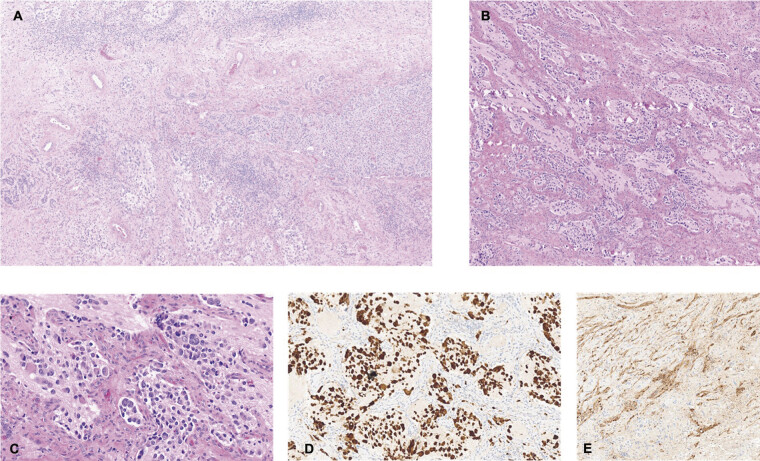
( **A–C** ) Histopathological findings of the resected specimen. The histopathological examination showed a pretreated adrenal neuroblastoma with 85% residual viable tumor. The residual viable tumor exhibited a morphology consistent with poorly differentiated and differentiating neuroblastoma embedded in extensive Schwannian stroma. ( **D** ) Anti-Hu immunohistochemistry, positive staining in neuroblasts. ( **E** ) Anti-S100 immunohistochemistry, positive staining in Schwann cells.

The histopathological examination showed a pretreated adrenal neuroblastoma with 85% residual viable tumor represented by poorly differentiated and differentiating neuroblastoma embedded in extensive Schwannian stroma ( Fig. 5 ). The genomic profile indicated favorable biology.

The first follow-up evaluation (MRI and mIBG scintigraphy) revealed no evidence of macroscopical tumor residual. At the 6-month follow-up, the patient remains off therapy, shows no evidence of disease recurrence, and continues regular clinical and imaging surveillance ( Video 1 ).

## Discussion

Surgery remains the fundamental tool in the treatment of localized neuroblastoma and retains a critical role even in challenging localized cases and in the context of metastatic disease. 1 2

The integration of VR images and ICG fluorescence in pediatric neuroblastoma surgery represents a significant advancement, offering a synergistic approach to address the challenges of these complex procedures. 4 5 6 7 8 VR enhances the surgeon's spatial awareness by incorporating preoperative imaging data into a dynamic, 3D framework, enabling precise tumor localization and improved navigation of intricate anatomical relationships. 4 5 6 7 8 This capability promotes safer and more accurate resections, reducing the likelihood of complications associated with the proximity of tumors to vital structures. 4 5 6 7 8 While the VR system, in this case, was used primarily for preoperative planning rather than intraoperative navigation, it differs from conventional 3D planning tools in several keyways. Unlike static 3D reconstructions viewed on a screen, the HMD provides an immersive, interactive environment that enables surgeons to explore the anatomy in three dimensions with intuitive hand controls. 5 21 22 23 The ability to manipulate structures—rotate, isolate, adjust transparency, and zoom—enhances spatial understanding and procedural planning. 5 21 22 23 This immersive approach supports team-based surgical rehearsals and interdisciplinary discussion, particularly in anatomically complex cases. 5 21 22 23 Although real-time intraoperative integration in the surgical field is not yet available, using VR in the preoperative phase represents a significant step forward in enhancing surgical precision and preparedness. 5 21 22 23

Simultaneously, ICG fluorescence complements VR by providing real-time vascular mapping, which is particularly beneficial in preserving critical structures such as the renal veins. 5 21 22 23 Its ability to dynamically visualize vascular anatomy could reduce the risk of accidental vessel injury and excessive bleeding, possibly contributing to improved surgical outcomes. 9 10 11 12 13

In this case, VR-guided navigation allowed the surgical team to anticipate the tumor's spatial relationship with major vessels, leading to a more cautious dissection plane. Intraoperatively, ICG fluorescence was instrumental in confirming the patency and integrity of surrounding vasculature, helping to prevent inadvertent vascular injury.

However, despite these benefits, certain limitations hinder the full potential of these technologies. While the current case involved a tumor displacing but not encasing major vessels such as the IVC and renal veins, it is important to note that many intermediate- and high-risk neuroblastomas present with a much more complex vascular involvement. In such cases, the tumor may envelop critical structures like the abdominal aorta, renal arteries, and veins, posing a significant surgical challenge even for experienced pediatric oncology surgeons. 6 7 8 In these more anatomically demanding scenarios, FGS using ICG may provide crucial intraoperative assistance by helping to identify and preserve vascular structures. 6 7 8 However, the effectiveness of ICG fluorescence is limited by its physical properties in the near-infrared window I (NIR-I, 700–900 nm), where the penetration depth is typically less than 1 cm. 24 This constraint may reduce its utility in visualizing deeply located or encased vessels before direct exposure. Consequently, while ICG-based FGS holds potential as a valuable adjunct tool, its current limitations must be acknowledged. Further improvements, such as alternative imaging strategies utilizing the NIR-II window (1,000–1,700 nm) or contrast agents with deeper tissue penetration, could enhance its intraoperative applicability in complex neuroblastoma resections. 24

Current VR systems in pediatric surgical oncology cannot superimpose 3D models directly onto the surgical field, requiring surgeons to recur to a preoperative VR navigation followed by intraoperative visual information relying on a secondary display. 5 21 22 23 This limitation highlights the need for technological advancements, such as real-time projection systems or HMDs, which could significantly enhance the practicality and precision of VR applications. An example of this approach was described by Ieiri et al., 25 who implemented an augmented reality navigation system for laparoscopic splenectomy in children using an optical tracking device to align preoperative CT images with intraoperative anatomy. Incorporating similar tracking and registration technologies in pediatric oncology surgery could enable precise overlay of 3D reconstructions during the procedure, moving beyond preoperative planning and toward fully integrated augmented reality guidance.

Similarly, while ICG fluorescence is invaluable for vascular imaging, its inability to delineate tumor margins remains a major drawback. 9 10 11 12 13 As demonstrated in this case, neuroblastoma tissues do not inherently retain ICG, making the technology unsuitable for direct tumor-specific imaging. 9 10 11 12 13 Addressing these limitations will require developing innovative solutions, mainly targeted imaging agents capable of binding specifically to neuroblastoma cells. 11 24 26 In recent years, several tumor-specific fluorescent agents have been investigated to overcome the limitations of ICG in neuroblastoma surgery. Among them, anti-GD2 antibodies conjugated to near-infrared dyes—such as anti-GD2-IRDye800CW—have demonstrated promising results in selectively labeling neuroblastoma cells in preclinical settings. 26 These targeted probes offer the potential to visualize tumor margins intraoperatively with greater precision and could significantly enhance fluorescence-guided resection.

Additionally, imaging in the second near-infrared window (NIR-II, 1,000–1,700 nm) is being explored for its deeper tissue penetration and improved signal-to-noise ratio, which may further advance intraoperative visualization in complex pediatric tumors. 24 These agents would enable precise visualization of neuroblastoma masses, even in cases where conventional imaging modalities fall short. 11 24 26 Though research in this field is still in its early stages, the potential to enhance intraoperative guidance and surgical precision is immense, especially for pediatric patients, where minimizing collateral damage is crucial.

While the integration of VR and ICG imaging enhanced intraoperative planning and visualization, the successful outcome also reflects other critical factors, including the patient's favorable response to neoadjuvant chemotherapy and the surgical team's expertise. The case highlights the importance of a multidisciplinary approach, where advanced imaging tools support—but do not replace—the clinical judgment and expertise of an experienced oncologic team. Widespread adoption of these technologies will require interdisciplinary collaboration among surgeons, oncologists, engineers, and researchers. To ensure their broader implementation, standardized protocols must be established, these tools must be incorporated into surgical training programs, and their efficacy must be validated through rigorous clinical trials. Additionally, large-scale multicenter studies will be critical to evaluating their impact on surgical outcomes, complication rates, and long-term prognoses.

As these innovations continue to evolve, they can significantly improve the surgical management of pediatric tumors. By enabling safer and more precise resections, these technologies could improve clinical outcomes and reduce the physical and emotional burden of surgery on young patients and their families. While this case illustrates the potential of VR and ICG fluorescence imaging to support surgical planning and safety, further studies and larger series are needed to validate these findings and establish standardized protocols for their integration into pediatric oncologic practice.

## Conclusion

This case highlights the potential of combining VR and ICG fluorescence imaging to enhance anatomical visualization and vascular mapping during complex pediatric neuroblastoma surgery. While these technologies contributed to surgical precision and safety, limitations such as the lack of tumor-specific fluorescence underscore the need for further advancements, particularly in targeted imaging agents. Continued innovation and clinical validation are essential to realize their role in pediatric oncologic surgery fully.

## Notes

Importance for a Pediatric Surgeon This case highlights how virtual reality and ICG fluorescence-guided imaging can enhance anatomical understanding and intraoperative safety during complex neuroblastoma resections. These tools may support surgical decision-making and help minimize risks in anatomically challenging pediatric oncology cases.
